# Analysis of Anxiety Scale and Related Elements in Endodontic Patients

**Published:** 2007-04-01

**Authors:** Hengameh Akhavan, Payman Mehrvarzfar, Mahshid Sheikholeslami, Masomeh Dibaj, Shahrooz Eslami

**Affiliations:** 1 *Department of Endodontics, Dental School, Islamic Azad University of Medical Sciences, Tehran, Iran*; 2 *General Practitioner, Tehran, Iran*

**Keywords:** Dental Anxiety, Dental Phobia

## Abstract

**INTRODUCTION:** Anxiety of patients is one of the problems in dentistry which are considered in recent years, and it prevents them from having a treatment out of stress. This study was conducted to specify anxiety prevalence and related elements among endodontic patients.

**MATERIALS AND METHODS:** The study was conducted on 150 patients referred to Endodontic department of dental school of Islamic Azad University, using a cross sectional descriptive method in 2006. Using background characteristics, the patients were classified as a matter of age, sex, education and related factors such as previous dental visit, unfavorable experience in dental office, and the most prevalent cause of referring to dentist. In this regard, Dental Fear Survey (DFS), questionnaire was used and patients were divided in three groups of anxiety level. The results were analyzed using Chi-square and Fisher exact tests.

**RESULTS:** The findings showed highest anxiety scales among dental office referents were statistically significant for age group of 20-30, women, and under diploma education (P<0.05).

**CONCLUSION:** Improving the knowledge about causes of anxiety and its preventive methods are suggested to dentists. They should also provide treatments without annoyance and trauma.

## INTRODUCTION

Having fear of dental treatments is one of problems which are seriously considered in recent years. Anxious patients avoid regular appointments of dental clinics. Having control during their treatment is actually more difficult. Studies showed that approximately 50 percent of people are in fear of dentistry and 8 to 11 percent are severely anxious in dental offices, so they just refer to dentists with acute problems (1-3).

Many factors are mentioned as the causes of anxious in dental office. The common reason is “pain” which may be caused by previous painful experiences.

Injection and cavity preparation are anxious producing stimuli too. The most fearful operations in dental office are root canal treatment and extraction. In some cases, pain, inflammation, and night sleeplessness cause anxiety in patients. There are other effective factors such as family, relatives, and friends’ fear (3-5).

Being aware of anxiety prevalence in patients can be helpful in finding a solution for decreasing the problem. This can be achieved by Dental Beliefs Survey (DBS) and DFS questionnaires (6-7). The aim of this study was to answer the survey’s question and to overcome the contradictions specified the scale of anxiety prevalence among endodontic treating patients along with presenting in endodontics section of dental faculty Islamic Azad University in 2006.

## MATERIALS AND METHODS

The survey was conducted on 150 patients referred to Endodontic Department of dental faculty of Islamic Azad University, using a cross-sectional descriptive method. At first we justify the research plan for patients and then asked them complete questionnaires contain age, sex, education and related factors such as previous dental visit, and unfavorable experience in dentistry. The answers to the questions were classified as:

1) complete disagreement

2) disagreement

3) no opinion

4) agreement

5) complete agreement.

The scores were registered.

The grades were 5 to 1, respectively as the most to less anxiety. The total grade of each answer sheet was a criterion to put subjects in a specific category from three domains of major, medium and minor anxiety. The results were collected, classified and analyzed using statistical software, Chi-square and Fisher exact test.

**Table 1:** Prevalence of anxiety scale according to the factors

**Anxiety Scale** **Demographic Factors**		**Mild**	**Moderate**	**Severe**	**Total**	***p*** **-value**
Age	20-30	6 (14/3)	27 (64/3)	9 (21/4)	42 (100)	P<0/05
	30-40	42 (58/3)	24 (33/3)	6 (8/4)	72 (100)	
Sex	Over 40	9 (25)	18 (50)	9 (25)	36 (100)	P<0/05
	Woman	30 (3113)	48 (50)	18 (18/7)	96 (100)	
	Man	27 (50)	21 (38/9)	6 (11/1)	54 (100)	
Education	<diploma	30 (30/3)	51 (51/5)	18 (18/2)	99 (100)	P<0/05
	>diploma	27 (52/9)	18 (35/3)	6 (11/8)	51 (100)	

## RESULTS

One hundred and fifty patients including 96 women (64%) and 54 men (361Yo) with the age of 35.2 ± 5.1 years were assessed.

Table 1 shows that the highest scales of medium anxiety belong to age groups of 20-30.

Chi- square test showed a statistical Significantdifference between age groups (P<0.05).

Women experienced higher scale of medium anxiety (50%) than men (38.9%) (P<0.05).

Highest medium anxiety scale belongs to under-diploma people and the highest minor anxiety belongs to high educated ones, 51.5% and 52.9%, respectively which was a significant statistical difference according to Chi-square (P<0.05).

Table 2 shows that the highest medium anxiety level belongs to patients with under and above 6 years of dental treatments' experience (44.7% and 50%t, respectively). Chi-square test shows no meaningful statistical difference.

It shows that the referents for check up have mostly minor anxiety (63.6%), while for dental problems the most anxiety was scaled as moderate (48.7%). There is a meaningful statistical difference according to Fisher exact test. The highest anxiety belongs to patients with no or 1-5 previous experience of dental treatments (47.1 % and 46.7%, respectively). This is not a significant difference according to Chi-square.

**Table 2:** Prevalence of anxiety scale according to relevant factors

**Anxiety Scale** **Dental Visit**		**Mild**	**Moderate**	**Severe**	**Total**	**p-value**
First	< 6 years	48 (42/1)	51 (44/7)	15 (13/2)	144 (100)	P>0/05
appointment	> 6 years	9 (25)	18 (50)	9 (25)	36 (100)	
Chief	Check up	21 (63/6)	12 (36/4)	0	33 (100)	P>0/05
complaint	DP ^a^	36 (30/8)	57 (4817)	24 (20/5)	117 (100)	
Number of	0	15 (29/4)	24 (47/1)	12 (23/5)	51(100)	P>0/05
previous	1-5	15 (33/3)	21 (46/7)	9 (20)	45 (100)	
appointment	> 5	27 (50)	24 (44/4)	3 (5/6)	54 (100)	
Unfavorable	Yes	18 (28/6)	30 (46/6)	15 (23/8)	63 (100)	P>0/05
experience	No	39 (44/8)	39 (44/8)	9 (10/3)	78 (100)	

**a:** Dental Problem

Finally, it presents that the highest rate of medium and major anxiety belongs to those having unfavorable experiences of dentistry (46.6% and 23.8%, respectively). Chi-square test shows a meaningful statistical difference (P<0.05).

## DISCUSSION

This research shows that the highest anxiety scales of medium and major occur in younger ages. This finding is in agreement with those of kvale *etal. *(5) and Asnaashari *et al. *(7).

In this study women reveal more anxiety than men, and this result is in agreement with Asnaashari, Tabrizizadeh, and sharet studies (6-8).

This research showed that more education leads to lower anxiety of dentistry. This is in agreement with Asnaashari's findings (7).

In this research, no relation between anxiety severeness and the first visit of dentistry is seen which confirms Tabrizizadeh's research (8).The findings showed that the highest anxiety is found in those who face acute toothaches. The same result is gained by Asnaashari research (7).

Surveys showed that there is no statistical Relation between anxiety scales and the number of dental visits. They are in conformity with findings of others (6-8).

Assessments of current study revealed noticeable results as follow:

1- Women reveal more anxiety over treatment than men.

2- Unfavorable experience of dentistry may produce fear and anxiety in patients.

3- Patients with acute problems in their teeth, feel more anxiety.

## CONCLUSION

Dentists are suggested to improve their knowledge about the causes of anxiety in patients and its eliminating methods.

Moreover, they should provide dental treatments.
